# Genetically-Directed, Cell Type-Specific Sparse Labeling for the Analysis of Neuronal Morphology

**DOI:** 10.1371/journal.pone.0004099

**Published:** 2008-12-31

**Authors:** Thomas Rotolo, Philip M. Smallwood, John Williams, Jeremy Nathans

**Affiliations:** 1 Department of Molecular Biology and Genetics, Johns Hopkins University School of Medicine, Baltimore, Maryland, United States of America; 2 Department of Neuroscience, Johns Hopkins University School of Medicine, Baltimore, Maryland, United States of America; 3 Department of Ophthalmology, Johns Hopkins University School of Medicine, Baltimore, Maryland, United States of America; 4 Howard Hughes Medical Institute, Johns Hopkins University School of Medicine, Baltimore, Maryland, United States of America; Tel Aviv University, Israel

## Abstract

**Background:**

In mammals, genetically-directed cell labeling technologies have not yet been applied to the morphologic analysis of neurons with very large and complex arbors, an application that requires extremely sparse labeling and that is only rendered practical by limiting the labeled population to one or a few predetermined neuronal subtypes.

**Methods and Findings:**

In the present study we have addressed this application by using CreER technology to non-invasively label very small numbers of neurons so that their morphologies can be fully visualized. Four lines of *IRES-CreER* knock-in mice were constructed to permit labeling selectively in cholinergic or catecholaminergic neurons [*choline acetyltransferase* (*ChAT*)*-IRES-CreER* or *tyrosine hydroxylase* (*TH*)*-IRES-CreER*], predominantly in projection neurons [*neurofilament light chain* (*NFL*)*-IRES-CreER*], or broadly in neurons and some glia [*vesicle-associated membrane protein2* (*VAMP2*)*-IRES-CreER*]. When crossed to the *Z/AP* reporter and exposed to 4-hydroxytamoxifen in the early postnatal period, the number of neurons expressing the human placental alkaline phosphatase reporter can be reproducibly lowered to fewer than 50 per brain. Sparse Cre-mediated recombination in *ChAT-IRES-CreER;Z/AP* mice shows the full axonal and dendritic arbors of individual forebrain cholinergic neurons, the first time that the complete morphologies of these very large neurons have been revealed in any species.

**Conclusions:**

Sparse genetically-directed, cell type-specific neuronal labeling with *IRES-creER* lines should prove useful for studying a wide variety of questions in neuronal development and disease.

## Introduction

A central organizing principle in all nervous systems is the division of neurons into different classes based on their distinctive dendritic and/or axonal morphologies. This principle was first appreciated over a century ago following the systematic analysis of individual neuronal morphologies in Golgi stained preparations [1]. Over the past several decades, methods for visualizing the morphologies of individual neurons have been developed that are based on intracellular injection of tracers such as horse-radish peroxidase (HRP), neurobiotin or biocytin, and dextran-conjugated fluorescent dyes, or bombardment with particles coated by carbocyanine dyes [2]–[5]. Labeling by intracellular injection has the virtue that the micro-pipette used for cell filling can also be used to characterize the neuron electrophysiologically, thereby generating a dataset that links morphological and physiological properties [e.g. ref. 6].

A second general strategy for visualizing neuronal morphology relies on the selective expression of genes coding for enzymes or fluorescent proteins. These genes can be introduced acutely into target neurons by viral infection, electroporation, or particle bombardment [7]–[10]. Although these methods are limited by the requirement for mechanical access to the neurons of interest and by the stochastic nature of cell targeting, they have the virtue of being relatively rapid and applicable to virtually any experimental animal. Alternatively, reporter genes can be introduced into the germline or into fertilized embryos in those model organisms amenable to such manipulation (nematodes, Drosophila, zebrafish, Xenopus, or mice). Among germline techniques for cell marking, ‘mosaic analysis with a repressible cell marker’ (MARCM) in Drosophila, and its cousin ‘mosaic analysis with a double marker’ (MADM) in mice, are conceptually the most general as they produce a sparse collection of cells distinguished by the exchange of a pre-defined chromosome arm that can carry an arbitrary set of genetic markers [11], [12].

In the mouse, genetically directed neuronal labeling has typically been achieved by the selective expression of a fluorescent protein or enzyme under the direct control of a cell-type-specific promoter or under the indirect control of a pharmacologically-regulated fusion between Cre recombinase and a mutated estrogen receptor ligand-binding domain [CreER; 13]–[16]. Several implementations of the sparse labeling approach have taken advantage of the serendipitous observation that transgenes driven by the Thy-1 promoter often show strong position effects that restrict expression to small subsets of neurons, with the cell type and labeling density being distinctive for a given transgenic line [17]–[19]. Other implementations have used BAC transgenes to produce fluorescent proteins or Cre recombinase in a cell type specific manner [20], [21].

The mouse studies reported thus far have not applied genetic labeling technologies to the morphologic analysis of neurons with large and complex arbors, an application that requires extremely sparse labeling and that is only rendered practical by limiting the labeled population to one or a few predetermined neuronal subtypes. In the present study we have addressed this application by developing and characterizing a series of mouse lines that express CreER in distinct neuronal subsets and we have used these lines in conjunction with a plasma membrane-anchored alkaline phosphatase reporter to visualize the morphologies of large CNS neurons.

## Results

### Construction of *IRES-CreER* knock-in lines

**Figure 1:**
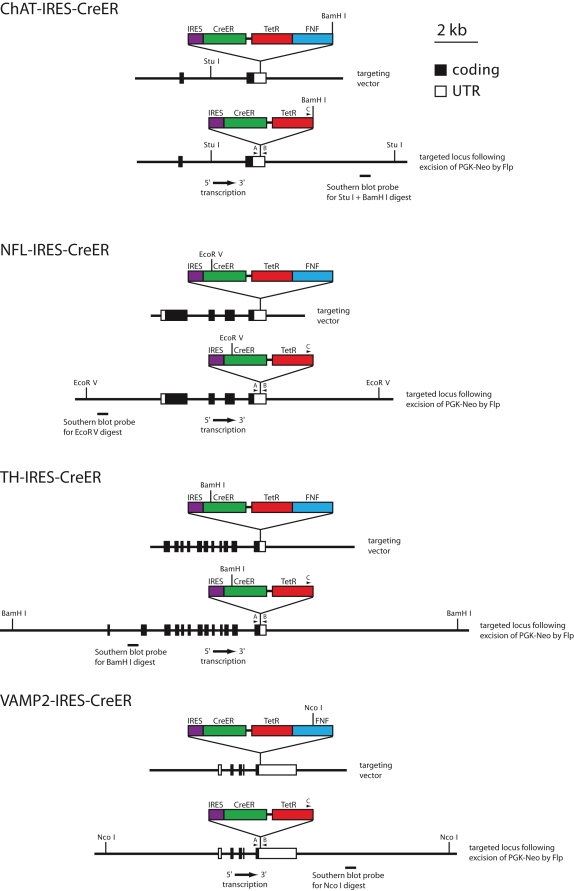
Restriction maps of the targeting constructs, targeted alleles, Southern blot probes, and PCR primers for *IRES-CreER* knock-in alleles at the *ChAT*, *NFL*, *TH*, and *VAMP2* loci. For each knock-in allele, a cassette consisting of an *IRES-CreER*, the pBR322 Tetracycline resistance gene (*TetR*), and an Frt-flanked phosphoglycerate kinase promotor neomycin resistance gene (*FNF*) was inserted into the 3′ untranslated region of the target gene as indicated. The *PGK*-*neo* casette was subsequently removed in vivo by Flp recombinase. The sizes of the homology regions used for the targeting constructs were: *ChAT*, 9.5 kb; *NFL*, 7.0 kb; *TH*, 9.2 kb; and *VAMP2*, 9.1 kb. For Southern blot hybridization, the probes indicated beneath the maps of the targeted loci were used, and genomic DNAs from the four groups of targeted ES cells and germ-line transmitted mice were cleaved with the following restriction enzymes: *ChAT*, *Stu* I and *Bam*H I; *NFL*, *Eco*R V; *TH*, *Bam*H I; and *VAMP2*, *Nco* I.

To achieve sparse cell-type specific Cre-mediated recombination, we chose a strategy in which an internal ribosome entry site (*IRES*) and a CreER coding region were inserted into the 3′ untranslated region (UTR) of three genes that are expressed in defined subsets of neurons and one gene that is expressed in neurons and a subset of glia (Figure 1). For the former category we used the genes coding for choline acetyltransferase (ChAT; cholinergic neurons), tyrosine hydroxylase (TH; catecholaminergic neurons), and neurofilament light chain (NFL; projection neurons). For the latter category we used the gene coding for vesicle-associated membrane protein 2 (VAMP2).

For each of the four target genes, *IRES-CreER* sequences were inserted by homologous recombination in embryonic stem (ES) cells. We chose this approach rather than conventional or bacterial artificial chromosome (BAC) transgenesis because we presumed that a knock-in allele would have the greatest likelihood of precisely recapitulating the expression pattern of the endogenous gene. Although constructing gene-targeted mouse lines is more labor intensive than constructing BAC transgenic mice, it has the virtue that only one knock-in mouse line needs to be characterized for each construct. In contrast, in a transgenic approach, multiple lines are typically characterized for each construct to assess the effects of line-to-line variation in transgene structure, copy number, and integration site, as this variation can affect the expression level or lead to variegated expression. A potential disadvantage of the knock-in strategy is that the insertion of foreign sequences within the 3′ UTR could compromise the function of the target gene, for example by altering mRNA transport, stability, or translation efficiency. In the present instance, no phenotypic effects were apparent for any of the *IRES-CreER* alleles in the heterozygous state or for the *ChAT-IRES-CreER*, *NFL-IRES-CreER*, or *TH-IRES-CreER* alleles in the homozygous state. However, homozygosity for the *VAMP2-IRES-CreER* allele appears to be lethal.

The strategy of inserting *IRES-CreER* sequences into the 3′ UTR places the exogenous DNA segment at a greater distance from the start site of transcription than would insertion of *CreER* sequences in the 5′ UTR, a design feature that, together with Flp-mediated excision of the Frt-flanked Phospho-glycerate kinase (*PGK*) promotor-neomycin resistance (*neo*) selectable marker (‘*FNF*’), should minimize perturbations in gene expression in the targeted allele (Figure 1). For the present set of four knock-in alleles, the distances between the start site of transcription and the point of insertion of the *IRES-CreER* cassette are: 57 kb (*ChAT*), 3.5 kb (*NFL*), 6.8 kb (*TH*), and 2.0 kb (*VAMP2*). As shown in Figure 1, the *IRES-CreER*-*FNF* cassette also includes a bacterial tetracycline resistance gene (*TetR*) 3′ of the CreER sequences, a selectable marker that facilitated the identification of bacterial colonies harboring the plasmids into which this cassette had been inserted.

### Cell type specificity of *IRES-CreER* expression

To characterize the cell type specificity of *IRES-CreER* expression, each targeted line was crossed to the *Z/AP* reporter line [22]. In the absence of Cre-mediated recombination, the *Z/AP* locus ubiquitously expresses a beta-galactosidase-neo fusion protein (beta-geo). Cre-mediated recombination excises the beta-geo coding sequence and simultaneously activates expression of human placental alkaline phosphatase (AP), a heat-stable enzyme that localizes to the plasma membrane via a GPI anchor [23], [24]. Previous work has shown that activation of the *Z/AP* reporter in neurons leads to the efficient accumulation of AP on individual axons and dendrites, which can be visualized by histochemical staining [14].

**Figure 2:**
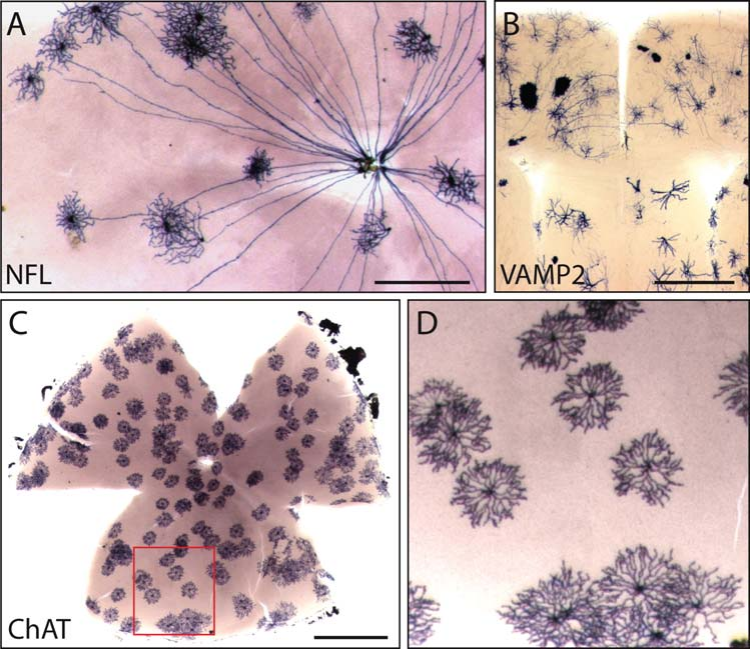
Cell-type specificity of Cre recombinase activity in mouse lines with different *IRES-CreER* knock-ins. The cell-type specificity of each line was tested by crossing to the *Z/AP* reporter and inducing sparse recombination with 4HT. A, In the retina, the *NFL-IRES-CreER* line exhibits Cre-mediated recombination in retinal ganglion cells, each of which has a single axon projecting to the optic disc, and in horizontal cells; the image shown here shows only retinal ganglion cells. B, in the brain, the *VAMP2-IRES-CreER* line exhibits Cre-mediated recombination in both neurons and glia. C and D, in the retina, the *ChAT-IRES-CreER* line exhibits Cre-mediated recombination exclusively in cholinergic (starburst) amacrine cells. The region boxed in C is shown at higher magnification in D. Scale bars: 0.5 mm in A and B; 1 mm in C.

Mice from the four *IRES-CreER;Z/AP* lines were treated with a single dose of 4-hydroxytamoxifen (4HT) during the first two postnatal weeks, and >1 month later tissues were harvested and the distribution of AP revealed by histochemical staining. The specificities of the *NFL-IRES-CreER* and *ChAT-IRES-CreER* lines were analyzed in flatmounts of adult retina. As seen in Figure 2A, AP-labeling in *NFL-IRES-CreER;Z/AP* mice is confined to retinal ganglion cells, each of which projects an axon to the optic disc, and less frequently to horizontal cells (not shown in Figure 2A), the two principal cell types in the retina that express neurofilament [25]. As seen in Figure 2C and D, AP labeling in *ChAT-IRES-CreER;Z/AP* mice is confined to cholinergic (starburst) amacrine cells, recognizable by their characteristic pinwheel morphology; these are the only cells in the retina that express choline acetyltransferase [26]. In the brain and retina, AP labeling in *VAMP2-IRES-CreER;Z/AP* mice is observed in a wide variety of neurons as well as in glia (Figure 2B and data not shown). Thus far, we have not observed *TH-IRES-CreER* mediated *Z/AP* recombination in the retina, a failure that we attribute to the paucity of dopaminergic amacrine cells [(450 cells per mouse retina; 27]. However, as described below, labeling in the brain in this line is consistent with expression in catecholaminergic neurons.

### Sparse genetic labeling for visualizing neuronal morphology

**Figure 3:**
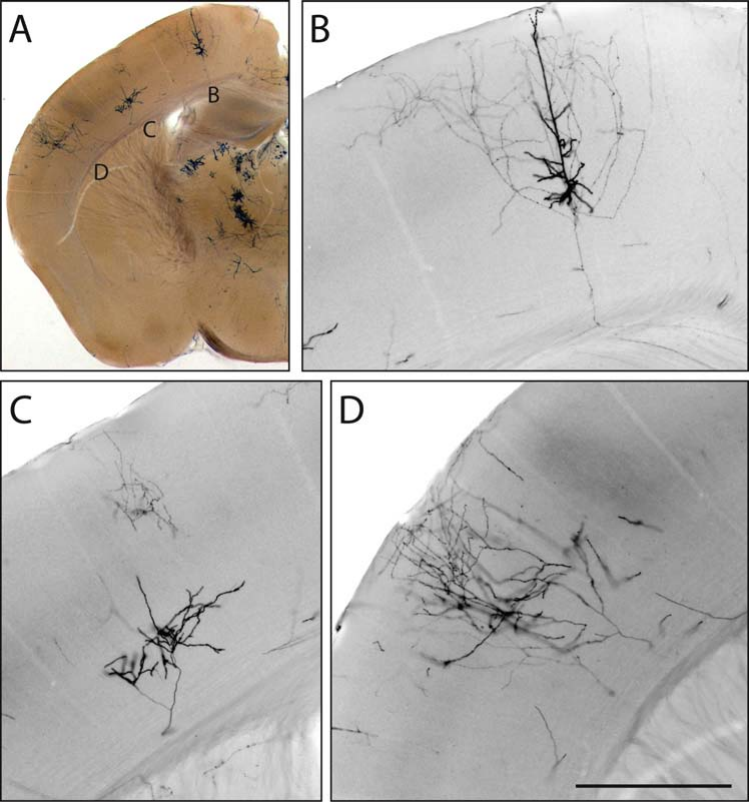
Sparse recombination permits visualization of non-overlapping neuronal processes. A, 200 um vibratome section from the brain of an *NFL-IRES-CreER;Z/AP* mouse injected at P0 with 0.2 mg 4HT. In this section, three large neurons (or parts of neurons) are labeled in one hemisphere of the cerebral cortex. B–D, enlarged bright field images at one Z-plane for each of the three neurons. Scale bar: 0.5 mm.

Figure 3A shows an example of sparse AP-labeling of cortical pyramidal neurons (or parts thereof) in a 200 um thick vibratome section from an *NFL-IRES-CreER;Z/AP* brain. Bright field images at a single focal plane are shown in Figure 3B–D, and demonstrate the clarity with which fine processes are visualized by AP histochemistry. The scarcity of labeled neurons is evident from the large regions of unlabeled cortex.

**Figure 4:**
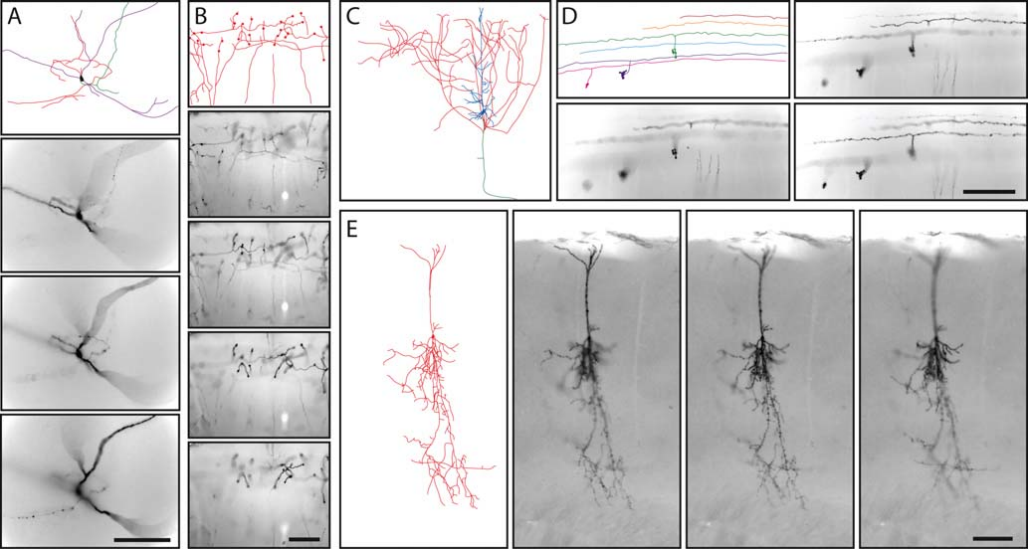
Tracing the morphologies of diverse AP expressing neurons from thick coronal sections of adult brains from various *IRES-CreER;Z/AP* mice. A, Multipolar neuron in the inferior cerebral cortex. B, Mossy fibers in the cerebellum. C, The cortical pyramidal cell shown in Figure 3B. D, Cerebellar granule cells. E, a compact cortical pyramidal cell. A–D are from *NFL-IRES-CreER;Z/AP* brains, and E is from a *ChAT-IRES-CreER;Z/AP* brain; the tracing of the cell in E is shown at lower magnification in Figure 5J. A, B, D, and E are traced from 300 um thick sections, and samples from the corresponding Z-series bright field images are shown. C is traced from a 200 um thick section; a sample from the Z-series image is shown in Figure 3B. Scale bars: 0.2 mm.

AP-labeled neurons can be fully reconstructed in 3-dimensions with NIH-Image or equivalent software, as demonstrated in a survey of retinal neurons [15]. In the present study, we have limited the reconstruction to a two-dimensional projection in the plane of each thick section, an image that we will refer to hereafter as a “tracing” rather than a “reconstruction” to distinguish it from a full three-dimensional reconstruction. This more limited analysis has the virtue of technical simplicity, while still illustrating (albeit, in projection) the principal morphologic features of the neurons under analysis. Examples of neuronal tracings together with samples of the corresponding Z-stack images from which they were obtained are shown in Figure 4. Figure 4C shows a tracing of the cortical pyramidal neuron in the 200 um section shown in Figure 3B. The remaining panels in Figure 4 - which show a multipolar cortical neuron (Figure 4A), cerebellar mossy fibers (Figure 4B), cerebellar granule cells with parallel fibers (Figure 4D), and a small cortical pyramidal neuron (Figure 4E) - are from sections of 300 um thickness. The tracings were prepared using Z-stacked images sampled at either 1 or 2 um intervals. As seen in Figures 3 and 4, AP intensity varies systematically with the type and location of neuronal processes, being heaviest in regions, such as the soma-proximal dendrites and apical process of pyramidal neurons, that are observed to be more intensely labeled in neurobiotin filling experiments [28].

For retinal ganglion cells, the *CreER;Z/AP* method labels dendrites, axons, and axon arbors (the latter in retino-recipient areas in the brain) in patterns that closely match those obtained by classical labeling methods such as diI and neurobiotin filling (Figure 2B; ref. 15; and T.R., T. Badea, and J.N., unpublished observations). As described below, among neurons with complex axonal arbors extending several millimeters, we observed no discernable variation in staining intensity within the axonal arbor. These data suggest that the >1 month delay between 4HT injection and sacrifice produces a roughly uniform distribution of AP over the entire extent of an axonal or dendritic arbor.

### Visualizing very large neurons in the mouse brain

The genetically-directed sparse labeling method described here has the potential to reveal the morphologies of neurons that would be difficult to visualize by classical cell filling methods due to their low abundance and/or large arbor size. To explore this potential, we have examined large neurons that were AP-labeled in mice carrying *IRES-CreER* inserted in the *ChAT*, *NFL*, or *TH* genes.

**Figure 5:**
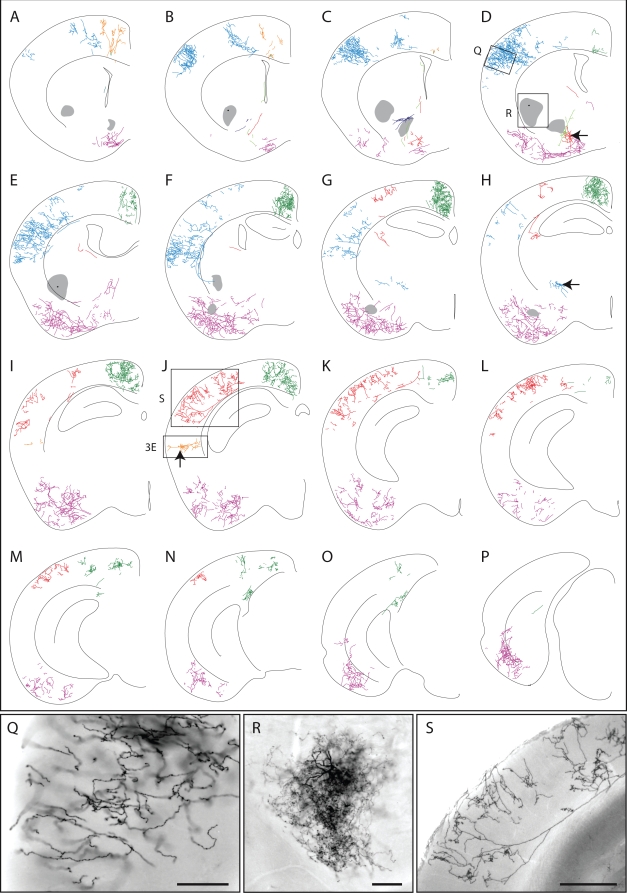
Morphologies of cholinergic neurons in an adult *ChAT-IRES-CreER;Z/AP* brain. Different neurons are color-coded. The mouse received injections of 4HT on P8 (1 mg), P21 (2 mg), and P28 (2 mg). A–P, tracing of neuronal processes from 16 serial 300 um coronal sections. Q–S and Figure 4E show bright field images of the AP-stained tissue corresponding to the boxed and lettered regions in D and J. All of the AP-stained neurons within the 16 sections are shown. Regions in which a high density of AP-labeled processes precluded accurate tracing are indicated by a semi-opaque grey zone, as shown for the cell in panel R. Cells bodies are indicated by black arrows. Scale bars: 0.2 mm in Q and R, 0.5 mm in S.

Figure 5A–P shows a single hemisphere from a *ChAT-IRES-CreER;Z/AP* brain in which all AP-labeled processes were traced in a series of 16 contiguous 300 um sections. Sixteen AP labeled neurons are color-coded. Sample images of the AP-stained tissue are shown in Figures 4E and 5Q–S, and correspond to the boxed and lettered regions in Figure 5D and J. The dendritic processes of four AP-labeled neurons in the basal forebrain were not clearly separable; these dendrites are colored purple in Figure 5C–E. Five other AP-labeled neurons, such as the one pictured in Figure 5R, have dendritic arbors that are too dense to accurately trace; these are represented as grey territories in Figure 5A–H. The tracings in Figure 5 show one large neuron with cortical arbors color-coded blue (Figure 5A–H) and a second one color-coded red (Figure 5B–N); each has an extensive axonal arbor in the cortex and a cell body in the basal forebrain with a small number of branching dendrites. For the two large neurons that are color-coded green and purple, the locations of the corresponding cell bodies could not be identified unambiguously. A striking observation from this set of tracings is that single cholinergic neurons possess arbors that ramify through nearly the full thickness of the cortex and extend across >2 mm^2^ of cortical surface. The cortical arbors of the neurons color coded in blue, green, red, and purple in Figure 5 span, respectively, ∼2.1 mm, ∼3.3 mm, ∼2.1 mm, and ∼3.9 mm along the rostro-caudal axis.

**Figure 6:**
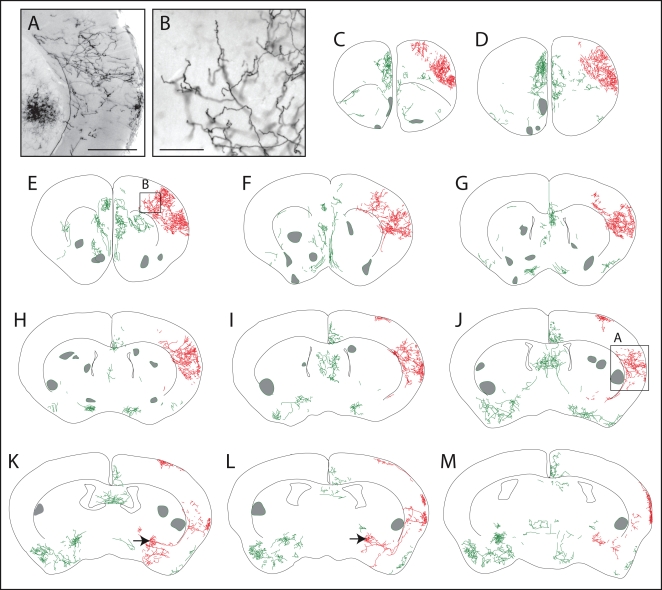
Morphologies of AP-labeled neurons in the rostral half of an adult *ChAT-IRES-CreER;Z/AP* brain. The mouse received injections of 4HT on P8 (1 mg), P21 (2 mg), and P28 (2 mg). A,B, bright field images of AP-stained tissue corresponding to the boxed regions in J and E, respectively. C–M, AP-labeled processes traced from eleven serial 300 um coronal sections. Two adjacent neurons with cell bodies in the basal forebrain (black arrows in panels K and L) and their large axon arbors in the cerebral cortex are shown in red. All other processes are shown in green. Regions in which a high density of AP-labeled processes precluded accurate tracing are marked by semi-opaque grey zones. Scale bar: 0.5 mm in A, 0.2 mm in B.

In the hemisphere shown in Figure 5, several contiguous zones encompassing ∼50% of the cortex are devoid of AP-labeled processes, and in the contralateral hemisphere of this brain ∼80% of the cortex is devoid of AP label (data not shown). A similar pattern of large and spatially clustered AP-labeled arbors was observed in other sparsely labeled *ChAT-IRES-CreER;Z/AP* brains (Figure 6). The spatial clustering of AP-labeled cortical processes implies that these processes derive from a very small number of neurons.

**Figure 7:**
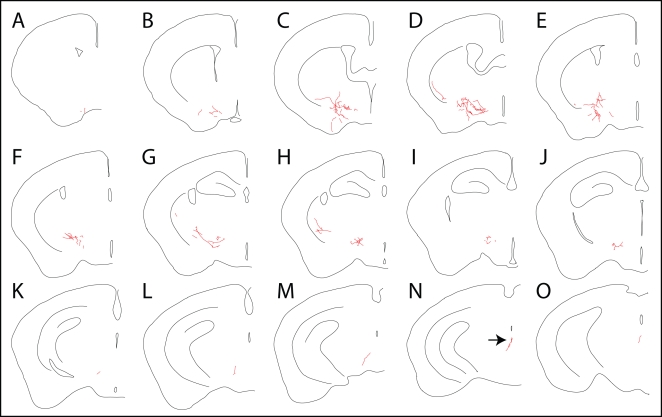
Tracing the morphology of a single AP-expressing neuron projecting from the periaqueductal gray to the lateral hypothalamus in an adult *TH-IRES-CreER;Z/AP* brain. A–O, Fifteen serial 300 um coronal sections. This is the only AP-expressing neuron in this hemisphere. The cell body is indicated by the black arrow in N.

Sparse neuronal labeling was also observed in the *TH-IRES-CreER;Z/AP* line following a single injection of 0.2 mg 4HT at P1. Figure 7 shows a single AP-labeled projection neuron in a series of 15 contiguous 300 um sections from a brain that contained only two AP-labeled cells; the second AP-labeled neuron was present in the contralateral hemisphere and is not shown here. The cell body of the AP-labeled neuron pictured in Figure 7 resides in the periaqueductal gray (arrow in Figure 7N), and its axon sends out branches along ∼3 mm of its >3.6 mm rostral trajectory. The most extensive branching is in the lateral hypothalamus (Figure 7C–F), a major target for projection neurons with cell bodies residing in the periaqueductal gray [29].

**Figure 8:**
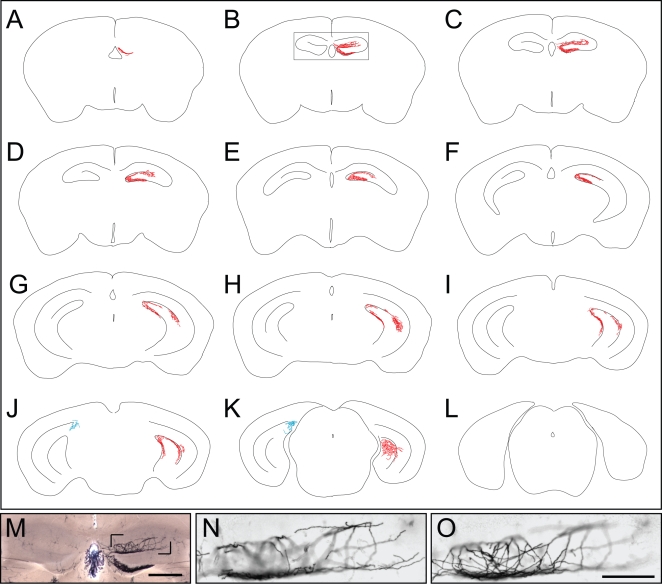
AP-labeled processes in the hippocampus in an adult *NFL-IRES-CreER;Z/AP* brain. A–L, Contiguous AP-labeled processes span eleven serial 300 um sections within the hippocampus. All of the AP labeled processes within the two hippocampi have been traced, but AP-labeled neurons outside of the hippocampus are not shown. M, anterior hippocampus corresponding to the boxed region in B shows unilateral AP-labeling. Some residual endogenous alkaline phosphatase activity is seen in the vasculature and choroid plexus. The cell body of this neuron has not been localized. N,O, Two images from a Z-stack of the region bounded by the pair of black corner marks in M. The processes of the AP-labeled neuron form a finely spaced meshwork near the surface of the molecular layer of the dentate gyrus. Scale bars: 0.5 mm in M, 0.2 mm in O.

In *NFL-IRES-CreER;Z/AP* mice that received a single 0.2 mg injection of 4HT at P0, the left and right hippocampi typically show highly asymmetric patterns of AP-labeling. For example, in the serial 300 um coronal sections in Figure 8, in which all of the AP-labeled processes in the hippocampus have been traced, almost all of the AP-labeling is unilateral. These AP-labeled processes comprise a contiguous and extensively ramifying arbor near the surface of the molecular layer of the dentate gyrus that encompasses the full rostro-caudal extent of the hippocampus. The absence of other AP-labeled processes over most of the hippocampus argues that the contiguous processes derive from a very small number of cells, and quite possibly a single cell. In a set of seven *NFL-IRES-CreER;Z/AP* brains with similarly sparse labeling, AP-labeling within the hippocampus was confined to a small number of compact pyramidal or granule neurons and/or to large arbors similar to the one shown in Figure 8. In this set, two, three, and two brains exhibited AP-labeling of the type shown in Figure 8 within both, only one, or neither hippocampus, respectively. Within each brain with AP-labeled hippocampi, the labeled processes occupied a contiguous territory. The spatial clustering of AP-labeled processes within the hippocampus is consistent with the inference that each contiguous AP-labeled arbor is likely derived from a single neuron.

The location, structure, and size of the large AP-labeled hippocampal arbors closely resemble the arbors of large hippocampal CA3 pyramidal neurons visualized by HRP, biocytin, and neurobiotin injection [30]–[32]. Although it is uncertain whether arbor filling was complete in all of these earlier studies, the different studies are in overall agreement with respect to the large size, extensive branching, and overall appearance of these arbors.

## Discussion

In this paper we have used the CreER technology to non-invasively label very small numbers of neurons so that their morphologies can be fully visualized. The four lines of *IRES-CreER* knock-in mice described here permit labeling that is selective for cholinergic or catecholaminergic neurons (*TH-IRES-CreER* or *ChAT-IRES-CreER*), that is enriched for projection neurons (*NFL-IRES-CreER*), or that is broadly targeted to neurons and some glia (*VAMP2-IRES-CreER*). We note that in the context of typical CreER-based tissue- and time-specific conditional knockout experiments, inefficient Cre-mediated recombination of target genes is generally considered undesirable [33]. By contrast, the strategy presented here exploits inefficient recombination to produce an extremely sparse collection of labeled neurons. As demonstrated here and in ref. 14, the number of recombined neurons can be reproducibly lowered to fewer than 50 per brain.

### General properties of the *CreER;Z/AP* method

For genetically-directed cell labeling to be useful for the analysis of neuronal morphology, Cre-mediated recombination following 4HT exposure should label only ∼1 in 10^4^–10^8^ neurons, depending on CNS location. In the system described here, this inefficiency arises from a combination of four factors: (1) the relatively inefficient translation of CreER coding sequences mediated by the *IRES* element [34]; (2) a serendipitous inefficiency of recombination at the *Z/AP* locus that renders it ∼1,000 fold less recombinogenic than other constructs in which a pair of loxP sites directs excision of an intervening segment of several kilobases [e.g., conditional knockout alleles at the *Frizzled5* locus [35] and at the *Brn3a* and *Brn3b* loci (T. Badea and J. Nathans, unpublished)]; (3) the dose and route of administration of 4HT; and (4) a progressive decrease in Cre-mediated recombination when 4HT is administered at later postnatal ages.

Sparse labeling also requires targeting of postmitotic cells to avoid generating clonally related clusters of neurons. For those CNS regions in which neuronal proliferation occurs prenatally (e.g. the cerebral cortex) this is readily achieved by 4HT injection at any postnatal age; for those regions in which neuronal proliferation continues postnatally (e.g. retina and cerebellum) 4HT injection can be delayed until ∼P8–P10. Furthermore, since expression of the *ChAT*, *NFL*, and *TH* genes is low or undetectable in proliferating progenitors, Cre-mediated recombination in the corresponding *IRES-CreER* knock-in lines should also be low in proliferating progenitors regardless of the time of 4HT injection. By contrast, in the case of *VAMP2-IRES-CreER;Z/AP* mice, 4HT injection at P2 produced clones of several hundred granule cells in the cerebellum (data not shown), implying expression of *CreER* from the *VAMP2* locus in proliferating granule cell progenitors.

Two properties of AP histochemistry are particularly useful for the morphologic analysis of large neurons: (1) efficient diffusion of the low-molecular weight histochemical substrates into fixed tissue and (2) the stability of the AP reaction product, which survives immersion in the tissue clearing agent benzylbenzoate∶benzyl alcohol (BBBA). These attributes of substrate and product permit processing and imaging of a small number of relatively thick sections (200–400 um thickness), thus minimizing the technically challenging alignment of neuronal processes between sections. We also note that the NBT/BCIP precipitate does not bleach or fade with visible light exposure and, if the tissue is stored in ethanol, the precipitate can be preserved for years with no apparent loss or diffusion. These attributes facilitate data collection over many imaging sessions. Although not utilized in the present study, AP activity can also be visualized by transmission electron microscopy via the production of a lead-phosphate precipitate, thus facilitating a correlation between light microscopic and ultrastructural analyses [27].

### Large size and complex morphology of basal forebrain cholinergic neurons

Cholinergic neurons with cell bodies located in the basal forebrain modulate cellular activity throughout the cerebral cortex, with wide-ranging physiologic effects. For example, cholinergic input modifies the response properties of neurons in primary sensory cortices, enhances the response to behaviorally significant stimuli in association areas, facilitates cortical control of motor output, and regulates vascular tone [36], [37]. Current evidence supports a model in which cholinergic neurotransmission modulates neuronal excitability but does not carry point-to-point information as glutamatergic and GABAergic transmission does [36].

Interest in the forebrain cholinergic system has been greatly stimulated by the observation that postmortem Alzheimer's disease brains exhibit a decrease in the number of cell bodies of cholinergic neurons in the basal forebrain and a parallel loss of enzymes involved in cholinergic transmission (e.g. ChAT) in the cerebral cortex [38]–[41]. Similar losses have also been reported in patients with Down syndrome, Parkinson's disease, and a variety of other progressive dementias [36], [42], [43]. These findings have led to the development of drugs to enhance cholinergic transmission as a strategy to partially ameliorate disease symptoms in patients with these disorders [44].

Sparse Cre-mediated recombination in *ChAT-IRES-CreER;Z/AP* mice permits the visualization of the full axonal and dendritic arbors of individual forebrain cholinergic neurons. To the best of our knowledge, this is the first time that the complete morphology of these cells has been revealed in any species. Earlier studies in which individual basal forebrain cholinergic neurons were labeled by injection of neurobiotin revealed their dendritic arbors but only incompletely filled their much larger axonal arbors [45]. The striking finding from the present study is that, for an individual cholinergic neuron, the axonal arbor is extremely large, with dense ramifications over a cortical surface area of >2 mm^2^ in the mouse. The large size of these cells is consistent with the simple idea that their loss in a variety of degenerative CNS diseases might reflect a correspondingly high metabolic burden and/or a high burden of toxic insults from amyloid deposition or reactive chemical species. Ideas along these lines have gained wide currency in the context of motor neuron disease and peripheral sensory neuropathies, with the enhanced susceptibility to disease in these contexts being related to the presence of an extremely long axon [46].

### Implications for visualizing neuronal morphology and sub-cellular structure

The most widely used methods for defining the morphologies of individual mammalian neurons involve fluorescent dye or neurobiotin filling by intracellular injection. In the simplest version of these methods, each neuron is identified solely on the basis of properties that are revealed after micro-electrode penetration: morphology, physiology, or both. However, this approach is extremely tedious if the goal is to target a particular neuronal subset, and it is impractical if that subset is rare.

A more efficient derivative of this method takes advantage of nondestructive approaches that reveal particular neuronal subsets in living tissue to guide intracellular tracer injection. For example, a particular subset of cell bodies can be visualized by retrograde labeling with fluorescent dextran, a method that has been widely used to analyze the morphologies of retinal ganglion cells that project to defined retinorecipient areas in the brain [e.g. ref. 2]. Alternatively, a subset of cell bodies can be visualized based on expression of a fluorescent protein marker [47]. The critical constraint in this more sophisticated approach to tracer injection is the requirement for optical access, making it practical for slice preparations, retinal flat mounts, or the more superficial layers of the cerebral cortex, but impractical for deeper brain regions. By contrast, the genetic alternative to tracer injection reveals neuronal morphologies based solely on sparse cell-type specific expression of a reporter.

The sparse labeling approach described here can be easily extended to Cre-control of other proteins. An especially interesting extension involves producing fluorescent fusion proteins that localize to subcellular structures such as synapses, mitochondria, or the cytoskeleton. This type of labeling has recently been reported following biolistic transformation of neurons in retina explants [48], [49]. Extending this analysis to neurons labeled in vivo by CreER-mediated activation of germline reporters would be particularly interesting in the context of CNS disease models.

## Methods

### *IRES-CreER* constructs

Genomic DNA segments encompassing the 3′ most exons of the mouse *ChAT*, *NFL*, *TH*, and *VAMP2* genes were isolated from an ES cell genomic library in bacteriophage lambda (a gift of Dr. Se-Jin Lee). For each knock-in allele, a cassette consisting of an *IRES-CreER*, the pBR322 tetracycline resistance gene (*TetR*), and a *Frt*-flanked phosphoglycerate kinase promotor-neomycin resistance gene (*FNF*) was inserted into the 3′ untranslated region within a genomic segment that had been subcloned into a plasmid carrying a Herpes Simplex Virus thymidine kinase gene. The *TetR* gene was included within the *IRES-CreER*/*FNF* cassette to facilitate identification of the final plasmid construct. The restriction site in the 3′ UTR into which the *IRES-CreER*/*TetR*/*FNF* cassette was inserted, together with the sequence of ten nucleotides on each side of the insertion sites are as follows. *ChAT*: *Sac* I, GGGCTGGGAGCTCCCTCTGA; *NFL*: *Pci* I, AGTCAATACATGTATAATTC; *TH*: *Acc*65 I, TGCATAGGGTACCACCCACA; *VAMP2*: *Bam*H I, CTCCCTTGGATCCGTGTGTG. Following electroporation into ES cells and positive and negative selection (G418 and gancyclovir, respectively), colonies were screened by Southern blot hybridization with ∼500 bp probes generated by PCR as shown in Figure 1. Karyotypically normal ES cells were used for blastocyst injection.

### Animal husbandry

Following germline transmission of the targeted allele, the *PGK*-*neo* cassette was excised by crossing to mice expressing Flp in the germline. Lines were maintained on a mixed Sv129×C57BL/6 background. *VAMP2-IRES-CreER* homozygotes are inviable, but the other three *IRES-CreER* lines can be maintained as homozygous stocks. All four lines have been deposited at the Jackson Laboratories and are freely available. Mice were handled and housed in accordance with the Johns Hopkins University Animal Care and Use Protocols and IACUC guidelines.

### Genotyping

As shown in Figure 1 and Table 1, PCR genotyping with a sense strand *TetR* primer (primer C), a sense strand 3′ UTR primer 5′ of the *IRES-CreER/TetR* insertion site (primer A) and a 3′ UTR antisense primer 3′ of the *IRES-CreER/TetR* insertion site (primer B) produces PCR products of different size for the untargeted allele and the knock-in allele, thus distinguishing homozygous WT, heterozygote, and homozygous knock-in genotypes. PCR was performed with Advantage 2 Polymerase (Clontech, La Jolla, CA) using 35 cycles of 30 seconds denaturation at 94°C, 30 second annealing at 60°C, and 1 minute elongation at 72°C. Rapid testing for the presence of the *Z/AP* allele was performed by X-gal staining of a small piece of tail.

**Table 1:** PCR Primers for genotyping.

*ChAT-IRES-CreER*
Primer A: ACTACTGGATCCAAGAGCTAGAGGCCCAACCAAGCCA
Primer B: ACTACTGAATTCTTCTGAATAGCTGGATGAGATAATC
Primer C: CGCATAGAAATTGCATCAACGCAT
Product sizes: A+B, 540 bp; B+C, 630 bp.
*NFL-IRES-CreER*
Primer A: ACTACTGAATTCAGAGGAGCAGGTGGCTAAGAAGAAAGA
Primer B: ACTACTGGATCCATTATTATTGTAAACATCTGTGTGATTCA
Primer C: CGCATAGAAATTGCATCAACGCAT
Product sizes: A+B, 333 bp; B+C, 497 bp.
*TH-IRES-CreER*
Primer A: ACTACTGGATCCAGTTTGACCCGTACACCCTGGCCAT
Primer B: ACTACTGAATTCGGAGACCTTTCCTTCCTTTATTGAGA
Primer C: CGCATAGAAATTGCATCAACGCAT
Product sizes: A+B, 400 bp; B+C, 570 bp.
*VAMP2-IRES-CreER*
Primer A: CTCGTCCTCCTTTCTCCCCCTCT
Primer B: AGAGAGGTGATGGGAACCTCAGG
Primer C: CGCATAGAAATTGCATCAACGCAT
Product sizes: A+B, 330 bp; B+C, 465 bp.

### 4HT injection and tissue processing

These procedures were performed essentially as described in ref. 14. In brief, 4HT was dissolved in ethanol, mixed with sunflower seed oil (Sigma, St. Louis, MO), centrifuged under vacuum to remove the ethanol, and delivered as a single intraperitoneal injection between P0 and P10 at a dose of 0.05–0.4 mg per mouse. After at least one month, the mice were anesthetized, and subjected to transcardiac perfusion with 4% paraformaldehyde in PBS. The brain was set in a block of 3% standard or low-melting point agarose in PBS and a complete series of 300 um coronal sections were cut with a vibratome. Brain sections and intact retinas in PBS were heated in a water bath at 65°C for 75 minutes to inactivate endogenous AP and, for those embedded in low melting point agarose, to melt the agarose surrounding the tissue; heat-inactivated tissues were then reacted in NBT/BCIP overnight at room temperature with gentle agitation. After washing and post-fixation, tissues were dehydrated with a graded ethanol series, and clarified in 2∶1 benzylbenzoate∶benzyl alcohol (BBBA). For long-term storage, tissue samples were returned to ethanol. AP-stained tissues can be stored in ethanol for several years with no change in appearance.

### Microscopy and neurite tracing

Images were captured on a Zeiss Stemi SV11 dissecting microscope equipped with OpenLab software and a Zeiss Axio Imager Z1 microscope equipped with a motorized stage and AxioVision software. Typically, 1 um or 2 um Z-steps were used to capture grey-scale bright-field or DIC images. As BBBA leads to tissue shrinkage of circa 30%, a complete Z-stack at 2 um intervals of what was initially a 300 um section can be captured with ∼100 images; the size of the resulting image stack is ∼180 Mbytes. For each vibratome section, a two-dimensional projection of AP-labeled neurites was obtained by manually tracing over the electronic images using an electronic stylus (Wacom, Saltama, Japan) and Illustrator CS2 software.
